# Rapid symptom control in neuroleptic malignant syndrome with electroconvulsive therapy: A case report

**DOI:** 10.3389/fpsyt.2023.1143407

**Published:** 2023-03-23

**Authors:** Lauren Katzell, Emily Beydler, Amílcar Silva dos Santos, Richa Vijayvargiya, Brent R. Carr

**Affiliations:** ^1^College of Medicine, University of Florida, Gainesville, FL, United States; ^2^Neuroscience Unit, CUF Tejo Hospital, Lisbon, Portugal; ^3^Mental Health Department, NOVA Medical School, Universidade Nova de Lisboa, Lisbon, Portugal; ^4^Universidade do Mindelo, Mindelo, São Vicente, Cape Verde; ^5^Department of Psychiatry, University of Florida, Gainesville, FL, United States

**Keywords:** electroconvulsive therapy, neuroleptic malignant syndrome, catatonia, amantadine, case reports, schizoaffective disorder

## Abstract

**Introduction:**

Neuroleptic malignant syndrome (NMS), thought to arise through dopamine antagonism, is life-threatening. While prompt diagnosis of NMS is critical, it may be obscured by other diagnoses, such as malignant catatonia, with overlapping, life-threatening symptoms. Initiation of dopamine-blocking agents such as antipsychotics and abrupt cessation of dopaminergic medications such as amantadine can precipitate NMS. Once NMS is suspected, deft medical management should ensue. Multiple case reports detail electroconvulsive therapy’s (ECT’s) effectiveness in the treatment of NMS. While this relationship is well-documented, there is less literature regarding comparative efficacy of ECT in the acute treatment of NMS-like states precipitated by withdrawal of dopamine agonists, such as amantadine.

**Case:**

We present a 52-year-old female with schizoaffective disorder bipolar type, with a history of a lorazepam-resistant catatonic episode the prior year that had responded to amantadine. She presented febrile with altered mental status, lead pipe rigidity, mutism, grasp reflex, stereotypy, autonomic instability, and a Bush-Francis Catatonia Rating Scale (BFCRS) of 24, suggesting malignant catatonia versus NMS. There was concern over a potentially abrupt cessation of her amantadine of which she had been prescribed for the past year.

**Interventions:**

Organic etiologies were ruled out, and a presumptive diagnosis of NMS was made with central dopaminergic depletion from abrupt dopamine agonist (amantadine) withdrawal as the suspected underlying etiology. After intravenous lorazepam and reinduction of amantadine failed to alleviate her symptoms, urgent ECT was initiated. Our patient received an index series of ECT of seven treatments. After ECT #1 she was no longer obtunded, after treatment #2 her symptoms of mutism, rigidity, stereotypy, and agitation showed improvement, and by ECT #3, the NMS had rapidly dissipated as evidenced by stable vital signs, lack of rigidity, and coherent conversation.

**Conclusion:**

Brisk identification of potentially life-threatening NMS and NMS-like states, including malignant catatonia, warrants a trial of ECT. ECT’s theoretical mechanisms of action coincide with the theoretical pathophysiology of the conditions. It is a viable and safe treatment option for reducing mortality. With prompt initiation of ECT, we obtained rapid control of a condition with a potentially high mortality.

## Introduction

Neuroleptic malignant syndrome (NMS) is a life-threatening condition characterized by fever, abnormal and widely fluctuating vital signs, “lead-pipe” rigidity, and elevated creatinine kinase (CK) (1). It is thought to arise through dopamine (DA) antagonism. The mortality rate of NMS is reported to be between 5 and 20%, with the rate increasing to up to 70% with complications such as aspiration pneumonia such that prompt diagnosis is critical (2, 3). With high symptom overlap, NMS is phenomenologically and physiologically related to malignant catatonia. The distinction, however, is that NMS is related to effective DA depletion such as antipsychotics (DA antagonists) or the withdrawal or rapid cessation of a dopaminergic agonist (4–6). Perhaps NMS lies on the most extreme continuum of severity of an underlying, diverse condition known as catatonia with its incompletely understood pathophysiological processes (7). Even with rapid cessation of DA agonists, NMS is not always induced, yet milder symptoms of NMS, mood symptoms, or motor abnormalities may be noted. Symptom clusters such as dysphoria, anxiety, fatigue, suicidal thoughts, orthostatic hypotension, and agitation are sometimes described as dopamine agonist withdrawal syndrome (DAWS).

In general, catatonia is often underdiagnosed, a result of its various presentations and variety of subtypes. The classic subtype, stuporous catatonia, is marked by the hallmark features of mutism, staring, immobility, withdrawal, posturing, and waxy flexibility (8). Contrast this to the subtype, excited catatonia, where psychomotor agitation, stereotypies, mannerisms, verbigeration, and echolalia predominate (9). And even more bewildering is periodic catatonia, involving fluctuations between excited and stuporous states; and delirious mania that manifests as typical mania, with signs of delirium, fever, and vital sign derangement (9). Most concerning, however, is malignant catatonia, first described in 1934 by Stauder, that is characterized by delirium, fever, stupor, and a mortality rate of over 50% (10).

Among patients seen on inpatient consultation-liaison psychiatry services, up to 6% may have catatonia (11). Due to the high mortality rates of NMS and malignant catatonia, it is essential to establish the diagnosis early. The variable presentations of catatonia and altered mental status are confounding and require a broad differential diagnosis. Moreover, serotonin syndrome has overlapping features with both catatonia and NMS, including abnormal vital signs and elevated CK (12). Linked to serotonergic activity (rather than DA), it manifests with hyperreflexia rather than muscle rigidity (12). Catatonia can be either primary, arising from an underlying psychiatric etiology such as a mood disorder or schizophrenia. Or it may be secondary to a toxic, metabolic, or neurological process. A useful, validated instrument aiding in the assessment is the Bush-Francis Catatonia Rating Scale (BFCRS) which assists in making the clinical diagnosis. The presence of 2 of 14 screening items is needed for diagnosis (8, 13). And a list of 23 items is provided to scale symptom severity. The DSM-5-TR provides an alternative metric and requires 3 of 12 diagnostic criteria (14). Given only 2 items are needed for a diagnosis of catatonia, BFCRS will be positive in patients with NMS, due to symptom overlap.

Malignant catatonia and NMS are similar conditions. The key differentiator is that NMS is precipitated by dopamine antagonism or by withdrawal of a dopaminergic agent (15). This contrasts with malignant catatonia arising from a primary underlying condition. In this sense, NMS might be considered as having a more iatrogenic origin—such as the addition of a DA antagonist or the removal of a DA agonist. Regardless of whether NMS is considered an iatrogenic malignant catatonia or merely the periphery on a continuum of catatonia, once either is suspected, deft medical management should ensue. ECT affords one of the fastest treatment responses within psychiatry for catatonia and a similarly rapid response is detailed in multiple case reports for the treatment of NMS due to antipsychotic use (15, 16). While its effectiveness is well-documented, there is less literature regarding efficacy or the speed of response to ECT for the acute treatment of NMS when it arises from DA agonist withdrawal, such as amantadine withdrawal.

## Case description

We present a 52-year-old female with schizoaffective disorder bipolar type, cutaneous lupus erythematous, rheumatoid arthritis, chronic kidney disease stage III, and seizure history requiring no antiepileptic medication. She presented to the emergency room febrile, with altered mental status, lead pipe rigidity, mutism, grasp reflex, stereotypies, autonomic dysfunction, tachycardia (110 beats per minute), hypertension (systolic blood pressure of 180 mm Hg). Creatine kinase and white blood cell counts were within normal limits. Within 48 h of admission, she experienced acute hypoxic respiratory failure and unspecific seizure activity requiring intubation. She received 2 mg intravenous lorazepam and a 30 mg/kg loading dose of levetiracetam with 500 mg every 12 h. This was complicated by aspiration, presumed to be from either rigidity or seizure, that led to MRSA pneumonia being treated with intravenous antibiotics. However, motor rigidity and autonomic abnormalities remained unchanged despite resolution of her pneumonia.

EEG monitoring revealed multiple head and arm shaking events; however, the recording did not have an electrographic correlate, wherein non-convulsive seizures were ruled out. Brain MRI was unrevealing. Lupus cerebritis and meningitis were ruled out through serologic testing, urosepsis was ruled out with urinalysis. The patient was subsequently extubated after being deemed able to protect her airway.

Her BFCRS was 24. Her current home medications included hydroxychloroquine 200 mg daily, prednisone 5 mg three times daily, amantadine 100 mg daily, venlafaxine extended release 75 mg daily, and lamotrigine 100 mg nightly. Of concern was her previous catatonic episode 1 year prior that had failed to respond to a four-week course of lorazepam that had been titrated to divided doses of 9 mg daily. However, that previous episode of catatonia remitted rapidly after initiation of amantadine. Unfortunately, further details regarding the treatment course of that episode are unknown.

Neuroleptic malignant syndrome vs. malignant catatonia were both high on the differential diagnoses. The emergency room had given a report of a rapid reduction in her amantadine, suggesting the potential of disruption in central dopaminergic activity, such that a presumptive diagnosis of NMS was made. The patient’s home amantadine was restarted. However, she showed only minimal, partial improvement in rigidity following amantadine resumption, and continued to exhibit severe catatonic symptoms.

After failing to improve with both intravenous lorazepam 4 mg four times per day and re-initiation of amantadine, an urgent index series of ECT was initiated once legal consent obligations were met. On the morning of her first ECT (now hospital day 12), she remained obtunded and continued to exhibit lead pipe rigidity, mutism, stereotypies, a grasp reflex, tachycardia, and hypertension. Bitemporal electrode placement was selected to afford potentially the fastest response rate (17). ECT stimulus parameters prioritized low frequency over stimulation train duration which offer the most efficient means for seizure induction. The initial total charge dose was 379 mC (0.9A, 0.5 ms, 7.0 s, 60 Hz). Although only theoretical as quality indicators, device seizure quality data is listed for reference: time to peak coherence (6 s) and to peak power (8 s), with the maximum power at 6406 μV (2). Maximum sustained coherence was 91.8%. The seizure abruptly stopped at 31 s with notable post-ictal suppression. All the remaining ECT-induced seizures ranged between 26 and 31 s, and all were deemed efficacious in quality based on seizure morphology.

By 12 h post ECT #1 she was no longer obtunded, becoming alert for the first time since her admission 12 days prior. Her rigidity had also lessened. After ECT #2, her agitation and mutism ceased. She was able to follow commands, state her name, and engage in brief conversation. Her bilateral rigidity was notably improved. The following day post- ECT #2, she reported feeling “much better” have some recollection of her feelings of agitation on the previous days. The remainder of the ECT series (totaling #7) was without complication and well-tolerated. During mid- ECT series, her seizure threshold was suspected to rise based on seizure EEG morphology and duration. This led to subsequent total charge dose titrations with a final charge dose of 454 mC.

After the resolution of the presumptive NMS, our patient manifested symptoms consistent with her long-standing diagnosis of schizoaffective disorder. Despite improved orientation, symptoms included looseness of associations, response to internal stimuli, and auditory hallucinations such that she was transferred to a free-standing community psychiatric hospital for ongoing treatment. It was strongly recommended that she enter a continuation ECT taper, especially given her ongoing psychosis with potential need for an antipsychotic which was concerning given the recent episode of NMS.

## Timeline

**Figure 1:**
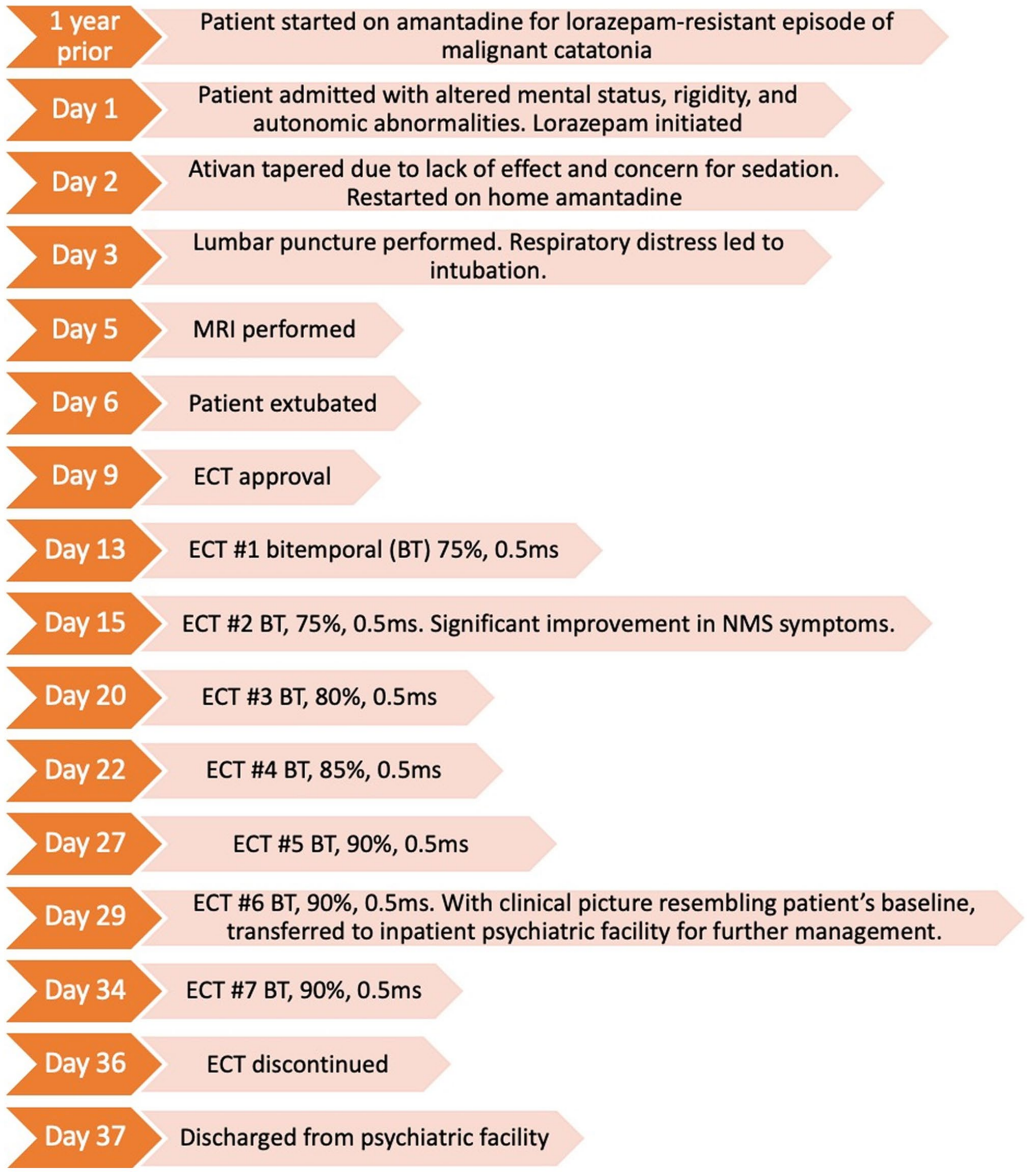


## Discussion

The pathophysiology of both catatonia and NMS are yet to be fully elucidated; however, it is thought to be related to a state of gamma-aminobutyric acid (GABA)-dependent dopamine (DA) depletion in various corticostriatal neural circuits (18). These networks include the motor circuit, anterior cingulate, orbitofrontal circuit, lateral hypothalamic connections, and lateral orbitofrontal circuit (18). Dopamine signaling in the striatum and paralimbic cortex is thought to be diminished as a result of reduced GABA-A inhibition of GABA-B (which decreases DA activity) (1). And benzodiazepines, such as lorazepam, a GABA-A agonist, are thought to improve catatonia by acting on this pathway (19). Conversely, reduced GABA-A inhibition of frontal corticostriatal tracts is associated with increased *N*-methyl-D-aspartate (NDMA) receptor activity, which is also thought to play a role in the pathogenesis of catatonia (20). Finally, amantadine and memantine, which antagonize such NMDA receptors, are sometimes used as adjunctive or alternative treatments for catatonia (21).

Patients with a history of malignant catatonia, such as our patient, are predisposed to higher rates of both a recurrence of catatonia and an incidence of NMS (22). Due to the high mortality risks associated with both malignant catatonia and NMS, consideration of ECT is warranted regardless of any ability to distinguish between these two conditions. For our patient, whose symptoms were presumed to be sequelae of dopamine agonist withdrawal (and whose symptoms persisted despite both re-initiation of amantadine and a trial of lorazepam), it was deemed critical to begin ECT for urgent symptom control (23, 24).

For our patient, a DA agonist withdrawal would have created a relative dopamine depletion. The symptoms arising from this depletion would be expected to respond to ECT (25, 26). It is theorized that ECT is efficacious for the treatment of NMS by increasing dopamine sensitivity and increasing dopamine release, in addition to increasing GABAergic, serotonergic, and noradrenergic transmission (25, 27–31). Limitations of this case report partially lie in the unverifiability in reports regarding her medication compliance, the timing of amantadine cessation, and the degree of her compliance prior to this reported abrupt cessation. However, even had this information been known, it would have mostly aided in diagnostic certainty rather than affording information on the potential efficacy of ECT. It is also unclear how much time was needed after amantadine re-initiation before a response is expected. After 12 days of having resumed amantadine, our patient had shown only an equivocal clinical improvement.

Although it is unknown if ECT was singularly causal in her rapid response, her NMS resolved only after initiation of ECT. It is theoretical that her meager response from amantadine was present with a potentially greater response emerging. Her severe symptomatology had continued despite amantadine re-introduction, yet she had at least not required intubation after the aspiration event. If any positive effects of amantadine were underway or were synergistic with the effects of ECT it was overshadowed by the robustness of her response and would have coincided with precisely the time of the first ECT treatment. It is also possible the effects were solely due to the ECT itself despite only one ECT treatment. Rapid changes occur within the brain during and after only one ECT. Same-day ECT responses are seen when treating catatonia (32); in depression, ECT can rapidly induce a mood change where polarity switches from depression to hypomania have been noted within 1–3 days of ECT of initiation of ECT 33–36.

However, the robustness of response does not aid in clarifying the issue as to whether this may be malignant catatonia instead, or that malignant catatonia and NMS are variations of the same phenomenon. One might even speculate that NMS arising from neuroleptic’s DA antagonism is different from NMS arising from DA agonist withdrawal—i.e., various NMS-like states. This is consistent with the idea that catatonia is a continuum. Moreover, case reports have suggested that rapid cessation of amantadine led to neuroleptic induced catatonia (NIC), again suggesting that catatonia and NMS are syndromes along a dopamine blockade continuum (26).

Even though our patient’s dose of amantadine was not particularly high (100 mg per day), its abrupt cessation is theoretically high enough to explain her presentation. There are reports of dose reductions of 100 mg every 2 days as unproblematic; however, the authors here suggest that dose reductions of 50 mg every second or third day (or slower), as being more prudent (37). Other reports have cited that even during a 2–3-day amantadine taper, such as with 200 mg or 300 mg, NMS cases have emerged (38, 39). These case reports also mention patients experiencing dopamine agonist withdrawal who have an array of unpleasant mood or motor symptoms yet do meet the full severity of an NMS—again suggesting a continuum.

Another consideration is that our patient’s home medication, lamotrigine, played a role in her symptoms. Early animal studies note that chronic treatment with lamotrigine regulates the expression of the inhibitory neurotransmitter GABA-A receptor (40). Moreover, lamotrigine also inhibits voltage-sensitive sodium channels, suppressing the release of glutamate (41). Only a few case reports have implicated lamotrigine as a contributor to NMS (although the underlying pathophysiology was unclear); and we noted only two that reported on its being implicated in isolation from any concurrent atypical antipsychotic use (42–44). These reports describe the occurrence of an NMS-like phenomena that occurred during a lamotrigine titration (or at least during the early weeks of its use), often in augmentation with an antipsychotic. However, our patient had been on lamotrigine for over a year. And, as with the amantadine, the actual medication compliance was unknown such that any contributory effects from lamotrigine for our case are also unknown. Regardless, should lamotrigine have been contributory to an NMS-like clinical picture, it would not have changed our decision to pursue ECT, which notably, increases GABAergic transmission.

Treatment refractory mood disorders typically respond to twice or thrice weekly ECT over a few weeks. The most optimal frequency for treating NMS is less well-established. Yet, given the potentially high mortality rate from NMS, daily ECT might even be justified when available. Our ECT treatments were delivered biweekly using bitemporal ECT electrode placement. It is unclear if more frequent ECT would have hastened her response but given the abrupt dissipation of her symptoms this seems moot.

This case also highlights the utility of ECT in patients who develop NMS secondary to withdrawal of dopaminergic agents, such as levodopa (in Parkinson’s Disease) and amantadine. The use of ECT should not be considered exclusive to psychiatric patients should symptoms arise suggesting an NMS-like state for such non-psychiatric patients. Moreover, BFCRS is an excellent tool for catatonia, but an underlying NMS should always be considered in the differential diagnosis given the large symptom overlaps. Given that malignant catatonia and NMS are both conditions with high mortality rates, it is critical to rapidly distinguish them from the problematic, but less pernicious, typical catatonia. This case further reinforces the notion that ECT is a safe and rapidly effective treatment for NMS. Here, ECT was effective in amantadine withdrawal-precipitated NMS, just as it has been shown to be for other NMS-like conditions. For antipsychotic induced NMS cases arising in patients with first order psychotic illnesses, it is likely they will continue to need dopamine antagonist medications. Given the well-documented benefits of a continuation taper of ECT for relapse prevention in mood disorders, it would be prudent to consider this for NMS patients as well. However, such guidelines are not well established.

In 200 words, describe the contribution of your manuscript to the research field. You should frame the research question(s) addressed in your work in the context of current knowledge, highlighting how the findings contribute to progress in your research discipline.

This case highlights the utility of ECT in treatment of NMS precipitated by amantadine withdrawal. ECT afforded rapid symptom reduction. This paper also importantly details the distinction between various forms of catatonia and the necessity of early recognition of malignant catatonia and the related condition, NMS. This case is applicable to physicians in multiple specialties, as many fields may encounter patients at risk for malignant catatonia and NMS, and awareness is paramount to swift intervention.

## Data availability statement

The original contributions presented in the study are included in the article/supplementary material, further inquiries can be directed to the corresponding author.

## Ethics statement

Ethical review and approval was not required for the study on human participants in accordance with the local legislation and institutional requirements. The patients/participants provided their written informed consent to participate in this study.

## Author contributions

BC, RV, and LK wrote the first draft of the manuscript. BC and LK wrote the final draft. All authors contributed to manuscript writing, editing, revision, and approval of the submitted version.

## Conflict of interest

The authors declare that the research was conducted in the absence of any commercial or financial relationships that could be construed as a potential conflict of interest.

## Publisher’s note

All claims expressed in this article are solely those of the authors and do not necessarily represent those of their affiliated organizations, or those of the publisher, the editors and the reviewers. Any product that may be evaluated in this article, or claim that may be made by its manufacturer, is not guaranteed or endorsed by the publisher.
